# Retraction: MicroRNA-300 inhibited glioblastoma progression through ROCK1

**DOI:** 10.18632/oncotarget.28716

**Published:** 2025-04-24

**Authors:** Fucheng Zhou, Yang Li, Zhen Hao, Xuanxi Liu, Liang Chen, Yu Cao, Zuobin Liang, Fei Yuan, Jie Liu, Jianjiao Wang, Yongri Zheng, Deli Dong, Shan Bian, Baofeng Yang, Chuanlu Jiang, Qingsong Li

**Affiliations:** ^1^Department of Neurosurgery, The 2nd Affiliated Hospital, Harbin Medical University, Harbin 150086, China; ^2^Harbin Medical University, Harbin 150086, China; ^3^Institute of Molecular Biology, Austrian Academy of Sciences, Vienna, Austria; ^*^These authors have contributed equally to this work

**This article has been retracted:** Oncotarget has completed its investigation of this paper. Several concerns were raised, including internal duplication and overlaps with unrelated papers from different institutions. Specifically, we found that Figure 3G contains overlapping invasion images of U87 and U251 cells transfected with control RNA. Additionally, the Figure 3G invasion image of U87 cells transfected with control RNA overlaps with the invasion image in Figure 3B from an unrelated 2014 paper [1] and duplicates an image found in paper [2], which was submitted and published concurrently. Furthermore, the western blot GAPDH and ROCK1 images in Figure 4F duplicate the GAPDH and RAB22A images published in 2015 in an unrelated and already retracted paper [3]. Finally, Figure 5A contains a GAPDH western blot also found in Figure 4A of an earlier published paper, which has been retracted [4], and in two slightly later published papers, which have also been retracted [5, 6]. We contacted Qingsong Li, the corresponding author, regarding these concerns. Unfortunately, the authors were unable to provide the transwell assay data or western blot images to support the data in the paper. In light of these facts, the Editorial decision was made to retract this paper. The authors did not respond to requests for signed documentation to confirm the retraction.

Original article: Oncotarget. 2016; 7:36529–36538. 36529-36538. https://doi.org/10.18632/oncotarget.9068
